# Migration Studies of Two Common Components of UV-curing Inks into Food Simulants

**DOI:** 10.3390/molecules24193607

**Published:** 2019-10-07

**Authors:** Miguel A. Lago, Raquel Sendón, Juana Bustos, María T. Nieto, Perfecto Paseiro Losada, Ana Rodríguez-Bernaldo de Quirós

**Affiliations:** 1 Department of Analytical Chemistry, Nutrition and Food Science, Faculty of Pharmacy, University of Santiago de Compostela, E-15782 Santiago de Compostela, Spain; miguelangel.lagocrespo@gmail.com (M.A.L.); raquel.sendon@usc.es (R.S.); perfecto.paseiro@usc.es (P.P.L.); 2 National Food Center, Spanish Agency of Food Safety and Nutrition, E-28220 Majadahonda, Spain; JBustos@mscbs.es (J.B.); MNietoG@mscbs.es (M.T.N.)

**Keywords:** migration, 4-methylbenzophenone, ethyl-4-(dimethylamino) benzoate, diffusion coefficient, partition coefficient

## Abstract

The Rapid Alert System for Food and Feed (RASFF) has reported many cases of different UV curing inks components in foodstuffs during the last few years. These contaminants reach foodstuffs mainly by set-off, their principal migration mechanism from the package. Under this premise, this work has tried to characterize the process of migration of two common UV ink components: a photoinitiator (4-Methylbenzophenone) and a coinitiator (Ethyl-4-(dimethylamino) benzoate), from the most common plastic material used in food packaging low-density polyethylene (LDPE) into six different food simulants. The migration kinetics tests were performed at four different common storage temperatures, obtaining the key migration parameters for both molecules: the coefficients of diffusion and partition. The migration process was highly dependent on the storage conditions, the photoinitiator properties and the pH of the foodstuff.

## 1. Introduction

In order to improve their quality and properties, most food packaging incorporates different additives into its structure as plasticizers, thermal and light stabilizers, slip additives or antioxidants. Consequently, the addition of these compounds enables the desired properties; nonetheless, the possible interactions between the packaging and the foodstuff should be considered from a food safety concern point of view. These interactions include mass transfer processes, commonly known as migration, which is defined as: “ mass transfer from an external source into food by submicroscopic processes” and it depends on several factors that can be summarized into four main factors: food, polymer, migrant and physical conditions: time and temperature [1,2].

Nevertheless, not only can additives migrate into foodstuffs. There is another wide group of compounds that could migrate from the food packaging: monomers, oligomers and their reaction products [3]. All of these molecules could reach the foods and depending on the migrant, they could represent a serious hazard for consumers’ health. To evaluate this hazard, migration experiments are usually performed, but they are expensive, time-consuming and, in some cases, complicated due to low migrant concentrations and/or due to the complexity of food matrices [3,4,5,6,7,8,9,10,11]. Food simulants are simple test media that mimic the behavior of real food and are allowed by the legislation. Their use simplifies the analysis.

To avoid these experiments, the current European legislation allows for the application of theoretical prediction models based on scientific evidence [12]. These models are based on Fick’s Second Law, which describes the migration process in the following equation:

(1) $$\frac{\partial C_{P}}{\partial t}=D_{P}\frac{\partial^{2}C_{P}}{\partial x_{2}}$$

where *C_P_* (mg∙kg^−1^) is the migrant concentration in the polymer at time *t* (s) and at distance *x* (cm), covered by the migrant from the origin; *D_P_* (cm^2^·s^−1^) is the coefficient of diffusion in the polymer.

One group of compounds which could migrate into foodstuffs, and which has received special attention in past years, are the UV printing inks components (e.g., photoinitiators). Printing inks are one of the seventeen groups of materials and articles included in the European Framework Regulation on materials and articles intended to come into contact with food EC 1935/2004 [13,14]. This regulation provides in its article 5 the possible adoption of “specific measures” for the different groups of materials, in order to ensure the protection of consumers’ health.

Since 2005 many notifications and alerts related to photoinitiators in foodstuffs have been reported through the Rapid Alert System for Food and Feed (RASFF) [15]. Therefore, the study of the processes involved in the migration of photoinitiators from the packaging into foodstuffs has become essential. The migration can occur by three ways; by diffusion from the printed layer to the food, by the set-off phenomena, namely, the transfer of substances when the printed external layer comes in contact with the internal layer during the storage of packaging materials in stacks or reels, and through the vapor phase [16,17].

Different studies have reported the presence of photoinitiators in various foods as a result of migration from the packaging. Van Den Houwe et al. (2016) [18] investigated the occurrence of several photoinitiators such as benzophenone, 4-(dimethylamino)benzophenone, ethyl-4-dimethylaminobenzoate, 2-isopropyl-9H-thioxanthen-9-one, 2,2-dimethoxy- 2-phenyl acetophenone and 4-phenylbenzophenone among others in dry foodstuffs including cereals, bread crumbs, pasta and rice acquired in the Belgian market. Samples were extracted using a Carrez-based method and analysed by LC-MS/MS in positive electrospray ionization mode. Benzophenone, ethyl-4-dimethylaminobenzoate and 2,2-dimethoxy- 2-phenyl acetophenone turned out to be the photoinitiators found in a greater number of samples and the highest amount detected was 0.262 mg/kg of benzophenone in a sample of rice.

GC-MS was the technique selected by Liu et al. (2016) [19] to determine photoinitiators in three types of milk, specifically, whole, semi-skimmed and skimmed. Solid-phase micro-extraction (SPME) was used as a sample preparation method. Benzophenone, 4-dimethylaminobenzoate and 2-isopropylthioxanthone were the photoinitiators detected in the samples.

The migration through the vapor phase has also been studied. A polyethylene enriched wax was used as a releasing source of photoinitiators to investigate the migration into dry foods by the gas phase [17].

Following this premise, in this work, two different components of UV printing inks were studied: 4-methyl benzophenone (4-MBP), a type II photoinitiator, and ethyl-4-(dimethylamino) benzoate (EDB), an amine used as a coinitiator. Low density polyethylene (LDPE) films were additivated with each substance to carry out the migration experiments at four common storage temperatures (4, 20, 40 and −18 °C) in different food simulants. To the best of our knowledge very limited information on migration of photoinitiators at freezing temperature has been reported in the literature. The effect of the temperature on the diffusion of photoinitiators was evaluated applying the Arrhenius equation. Diffusion and partition coefficients were estimated by fitting the experimental data with a mathematical model based on Fick’s Second Law [20].

## 2. Results and Discussion

### 2.1. Mathematical Modelling

Crank (1975) proposed various solutions for Eq. 1 depending on the scenario. If a plane sheet, in our case a LDPE sheet, is suspended in a stirred solution with a finite volume, a possible solution for a Polymer-Food system could be shown by Equation (2), which expresses the amount of migrant released from the polymer (*P*) into food (*F*) at time *t*: [4,20,21,22,23]

(2) $$\frac{m_{F,t}}{A}=C_{P,0}\rho_{P}d_{P}\left(\frac{\alpha }{1+\alpha }\right)\times \left[1-\overset{\infty }{\underset{n=1}{\sum }}\frac{2\alpha \left(1+\alpha \right)}{1+\alpha +\alpha^{2}q_{n}^{2}}\exp\left(-D_{P}t\frac{q_{n}^{2}}{d_{P}^{2}}\right)\right]$$

where α=1KPF VFVP and $K_{P/F}=\frac{C_{P,\infty }}{C_{F,\infty }}\frac{\rho_{P}}{\rho_{F}}$, *m_F,t_* is the mass of migrant from *P* into *F* after time *t* (μg); *A* is the area of *P* in contact with *F* (cm^2^); $C_{P,0}$, $C_{P,\infty }$ and $C_{F,\infty }$ are the concentrations of migrant in the *P* at *t* = 0, *t* = ∞ and the concentration of migrant in the *F* at *t* = ∞ (mg·kg^−1^); *ρ_P_* and *ρ_F_* are the densities of *P* and *F* (g·cm^−3^), *t* is the migration time (s), *d_p_* is the thickness of *P* (cm), *V_P_* and *V_F_* are the volumes of *P* and *F* (cm^3^), *q_n_* are the positive roots of the transcendent equation $tanq_{n}=-\alpha q_{n}$, *D_P_* is the diffusion coefficient of migrant in *P* (cm^2^·s^−1^), and *K_P/F_* is the partition coefficient of the migrant between *P* and *F*.

The experimental data obtained in the migration experiments from LDPE into the food simulants were fitted, with the proposed model based on Equation (2). With this purpose, the Solver function of the software Microsoft Excel 2010^®^ was used. The values of diffusion and partition coefficients (*D_P_* and *K_P/F_*) were determined for each migrant and food simulant at the different temperatures tested. These data are shown in Table 1 and Table 2, in addition, the Root-Mean-Square Error (RMSE %) was calculated. The RMSE showed values lower than 7.0% in all cases, except for the migration of 4-MBP into 50% ethanol (*v/v*) (RMSE < 9.0%). The good fitting of the experimental data with the model demonstrates that it can be used to predict the migration process of these migrants from LDPE to foodstuffs.

### 2.2. 4-MBP

This benzophenone derivative has a similar molecular weight to EDB, however, 4-MBP presents a higher log K_o/w_ value, being more lipophilic than the amine synergist. According to this value of log K_o/w_, 4-MBP, it is expected to have more affinity to lipophilic simulants such as 50% or 95% ethanol (*v/v*) than hydrophilic food simulants. The K_P/f_ values confirmed this fact ranging from 407.0 in 3% acetic acid (*w/v*) at 20 °C to values close to 1 in 95% ethanol (*v/v*) at 4 °C (Table 1).

**Figure 1:**
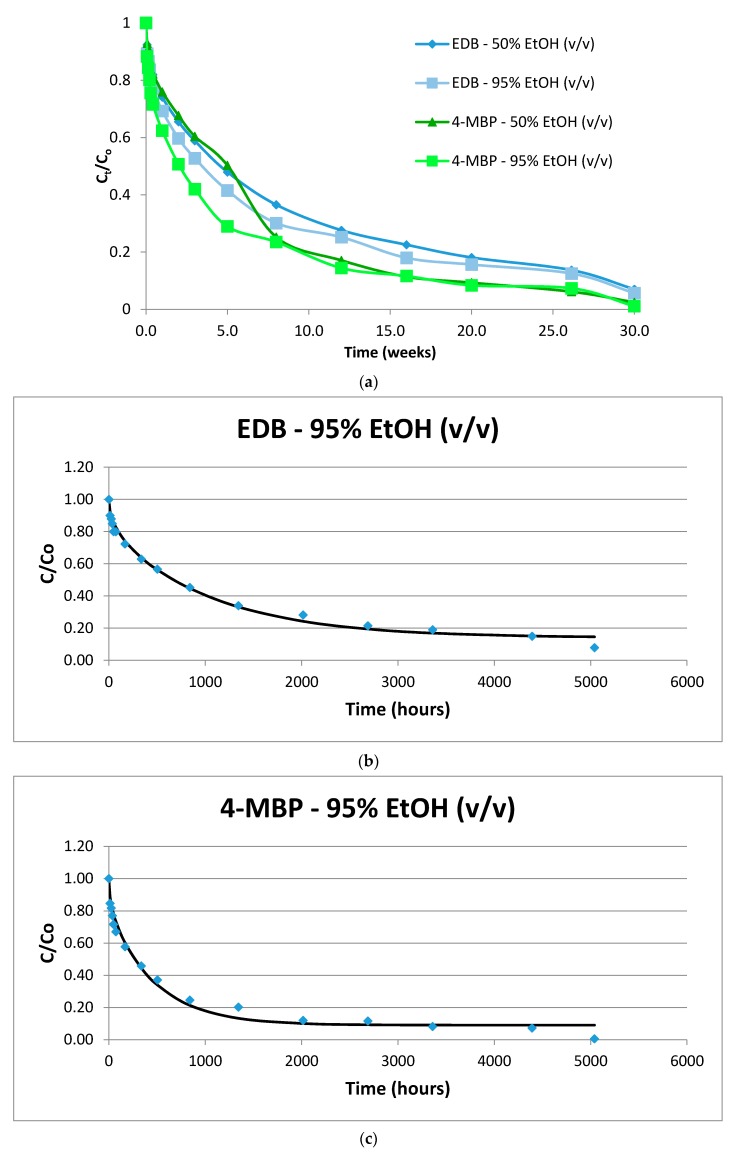
(**a**) Migration of 4-methyl benzophenone (4-MBP) and ethyl-4-(dimethylamino) benzoate (EDB) at −18 °C. C_o_: Initial concentration; C_t_: concentration at time t; (**b**) EDB 95% EtOH (*v/v*) (−18 °C); (**c**) 4-MBP 95% EtOH (*v/v*) (−18 °C).

As expected, higher temperatures led to higher diffusion coefficients in all food simulants without exception. Even freezing temperatures allowed for the migration of both compounds, as can be observed in Figure 1, presenting the lowest *D_P_* values (1.5 × 10^−12^) in 50% ethanol (*v/v*). On the other hand, the highest value of *D_P_* (3.1 × 10^−8^) was obtained at 40 °C, in 95% ethanol (*v/v*).

4-MBP is a photoinitiator derived from benzophenone (BP). In comparison with BP, the addition of a methyl group to the molecule increases the molecular weight from 182.22 to 196.24 and the log K_o/w_ from 3.18 to 3.69. These differences between both compounds affected the migration process as can be observed if these data were compared with those obtained in other works for BP [24]. Regarding to K_P/f_, from Table 1 it can be inferred that the slightly more lipophilic character of 4-MBP led to lower values of K_P/f_ as obtained for 95% ethanol (*v/v*), showing the tendency of the photoinitiator to migrate to lipophilic mediums rather than to remain in the film. On the contrary, the highest values were obtained in aqueous mediums (148–400) slightly higher than for BP [24].

The migration kinetics of EDB and 4-MBP in 95% and 50% ethanol (*v/v*) at −18 °C are presented in Figure 1a and the fitting curves in Figure 1b,c, respectively.

The *D_P_* values of both photoinitiators were similar in 10, 20, 50 and 95% ethanol (*v/v*); however, in water and 3% acetic acid (*w/v*), 4-MBP showed lower values than BP, particularly at high temperatures (3.2 × 10^−10^ and 4.1 × 10^−10^ for 4-MBP and 1.0 × 10^−9^ and 1.4 × 10^−9^ for BP). This last fact could be attributed to both parameters: the lower molecular weight and log K_o/w_ of BP compared to 4-MBP.

**Figure 2:**
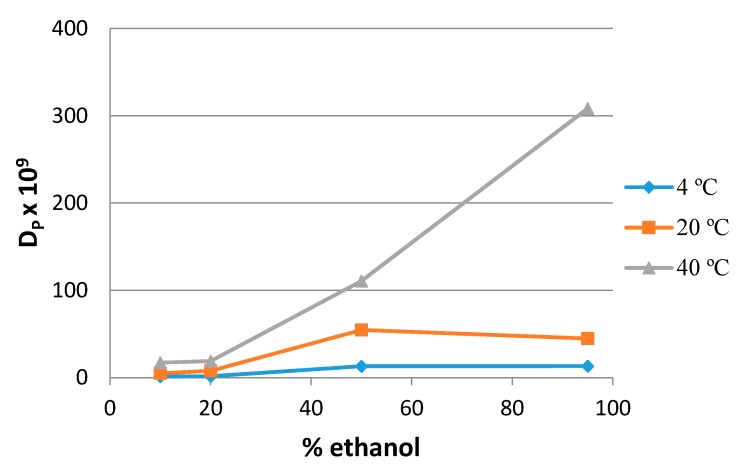
Relation between the 4-methyl benzophenone (4-MBP) diffusion coefficient (*D_P_*) and the percentage of ethanol of the food simulant.

Sanches-Silva et al. (2008) [22] reported a correlation between the percentage of ethanol and the diffusion coefficient values for BP, 1-Hydroxycyclohexyl phenyl ketone (HCPK) and 2-/4-Isopropyl-9H-thioxanthen-9-one (ITX). Additionally, in this work, 4-MBP shows a higher *D_P_* value at a higher percentage of ethanol in the food simulant [24]. This behaviour could be explained because of the swelling of the material, which led to a faster migration. Figure 2 represents the influence of the percentage of ethanol in the *D_P_* values at 4 and 40 °C, showing a linear relation (R^2^ = 0.9742) between both parameters, more evident at higher temperatures, (as was the case for BP and HCPK).

### 2.3. Ethyl-4-(dimethylamino) Benzoate (EDB)

The amine synergist EDB is a tertiary amine with a molecular weight similar to 4-MBP (193.24 against 196.24). As for 4-MBP, the values of *D_P_* were highly influenced by the temperature as expected, ranging from 5.5 × 10^−13^ in 50% ethanol (*v/v*) at −18 °C to 1.2 × 10^−8^ in 20% ethanol (*v/v*) at 40 °C. The *K_P/F_* values showed more affinity for fatty simulants (e.g., *K_P/F_* <1.4 for 95% ethanol (*v/v*)) than for hydrophilic ones (K_o/w_ values in water over 47.1) as the log K_o/w_ suggested.

Nevertheless, this coinitiator presented characteristic behaviour in 3% acetic acid (*w/v*). As can be reflected in Table 1, *K_P/F_* values obtained in the acidic food simulant were similar to those obtained in the more lipophilic food simulants such as 20% ethanol (*v/v*). However, the *K_P/F_* values in water were sensibly higher, ranging from 47.1 to 119.4. Food simulant 3% acetic acid (*w/v*) presented a pH close to 2.53 and, at this pH, approximately 72% of the migrant was in the protonated form (calculated by MarvinSketch 6.2, ChemAxon Ltd. Budapest, Hungary). This would explain the higher affinity for this food simulant. These *K_P/F_* values demonstrated that the pH of the food simulant was a key factor in the EDB migration process.

### 2.4. Diffusion Coefficient Linearity

Considering that the coefficient of diffusion depends on the temperature [11], the Arrhenius equation (Equation (3)) allows for the determination of the relationship between both factors

(3) $$lnD=-\frac{E_{A}}{R}\frac{1}{T}+lnD_{0}$$

where *D*_0_ is the pre-exponential factor (cm^2^∙s^−1^), which corresponds with the theoretical values of *D* at a temperature equal to infinite; *E_A_* is the activation energy (kJ∙mol^−1^); *R* is the ideal gas constant (8.31 × 10^−3^ kJ∙mol^−1^∙K^−1^); and *T* is the temperature (K).

The Arrhenius equation was applied to the diffusion coefficients obtained for EDB and 4-MBP into the different food simulants (Table 2). Taking into account the assumption that the coefficient of diffusion is dependent on the temperature, the linearity between lnD_P_ and 1/*T* from 4 to 40 °C was checked, obtaining acceptable R^2^ values (R^2^ > 0.95148 for 4-MBP and R^2^ > 0.91564 for EDB) in all food simulants. This fact allowed for the calculation of the diffusion coefficients of both molecules in this range of temperatures.

**Figure 3:**
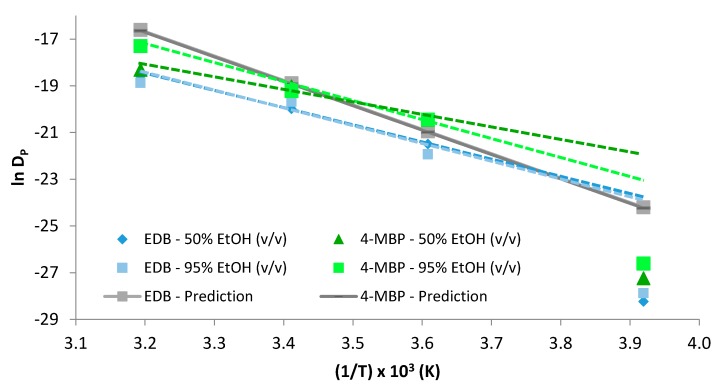
Application of the Arrhenius equation to estimate the relation between the obtained *D_P_* for ethyl-4-(dimethylamino) benzoate (EDB) and 4-methyl benzophenone (4-MBP) and the temperature. Dotted lines extrapolate the linearity estimated in the range 4–40 °C down to −18 °C

Figure 3 presents the application of the obtained *D_P_* to the Arrhenius equation in the range of temperature −18 to 40 °C in 50 and 95% ethanol (*v/v*). As discussed above, there was a linear relation between ln *D_P_* and 1/*T* from 4 to 40 °C; however, this linear relation was not observed between 4 and −18 °C. This figure shows how the experimental data obtained at −18 °C did not fit the linearity calculated from 4 to 40 °C, being the experimental *D_P_* values more than one order of magnitude lower than expected. These experimental results demonstrated that the migration from LDPE of both migrants continued until freezing temperatures (−18 °C); however, it was slower than expected and further studies should be performed in order to explain this behaviour.

### 2.5. Worst Case Prediction

Finally, a prediction of the “worst case scenario” of the migration of EDB and 4-MBP from LDPE was carried out. For that purpose, the equational approach based on the phenomenological derivations and statistical evaluation of experimental diffusion and migration data (Equation (4)) developed by Piringer (1994) was used [21,23,25].

(4) D∗P=104exp(AP−0.1351Mr23+0.003Mr−10454T)

with

(5) $$A_{P}={A^{\prime }}_{P}-\frac{\tau }{T}$$

where D*_P_ is the polymer specific upper-bound diffusion coefficient in cm^2^·s^−1^, *M_r_* is the relative molecular mass of the migrant in *D*, *A_P_* is a parameter that describes the behavior of the migrants in the diffusion, *A′_P_* is a polymer related parameter of diffusion, independent of the temperature and τ is a polymer specific “activation energy” parameter in K. In this case, the values for *A′_P_* and τ were 11.5 and 0, respectively for LDPE.

For a valid estimation of the worst case scenario, the D*_P_ values should have been higher than the *D_P_* obtained experimentally; however, Figure 3 showed that *D_P_* > D*_P_ for 4-MBP at 4 °C. Taking into account the linearity of *lnD_P_*, this representation allows the calculation of the temperature at which *D_P_* > D*_P_, being 16.9 °C in 95% ethanol (*v/v*), and 11.5 °C in 50% ethanol (*v/v*). Despite these results, further studies should be accomplished in order to confirm that this equation does not overestimate the diffusion coefficients of 4-MBP in 50% and 95% ethanol (*v/v*). Different sources of error could lead to a “non-overestimation” of the diffusion coefficients, the adjustment done by the mathematical model, a non-homogenous distribution of 4-MBP in the LDPE, the polymer thickness variation or possible interactions between the film and the food simulant [26].

## 3. Materials and Methods

### 3.1. Reagents and Standards

The UV printing inks compounds selected in this work: 4-MBP (CAS Registry No. 134-84-9; 4- methyl benzophenone) and EDB (CAS Registry No. 10287-53-3; Ethyl-4-(dimethylamino)benzoate), were purchased from Sigma-Aldrich (Steinheim, Germany). Their main properties are summarized in Table 3. Different food simulants were prepared by dilution of ethanol (absolute for analysis) and glacial acetic acid in distilled water. Solvents used in the chromatographic analysis were HPLC grade acetonitrile and ultrapure water from a Milli-Q filter system (Millipore, Bedford, MA, USA). All the reagents were purchased from Merck (Darmstadt, Germany).

### 3.2. Migration Test

Food simulants selected in this work were those in the Commission Regulation (EU) No 10/2011 (2011) [12]: 3% acetic acid (*w/v*), 10%, 20% and 50% ethanol (*v/v*). Instead of the conventional fatty simulant, vegetable oil, 95% ethanol (*v/v*) was used (it should be noted that for clarity throughout this entire document the term simulant is used for the conventional and the substitute simulants). Tests were also performed in water, in order to compare the data obtained in the current food simulant for hydrophilic foods: 10% ethanol (*v/v*) and the equivalent former food simulant, water, which is currently considered a hydrophilic food [27].

Migration tests were carried out at −18 °C, 4 °C, 20 °C and 40 °C. At −18 °C only migration assays in 50% and 95% ethanol (*v/v*) were performed since the other simulants were not a liquid at this temperature.

To carry out the migration tests, additivated films of low-density polyethylene (LDPE) were prepared by extrusion (at Gaiker, Zamudio, Spain) after mixing LDPE, Alcudia^®^ 2008F provided by Repsol-YPF (Madrid, Spain), with EDB or 4-MBP. The films were processed with a Polylab Haake Rheomex (Thermo Scientific, Waltham, MA USA) mono-screw extruder operated under the following conditions; screw speed 20 rpm and temperature in heating zones were 185, 205, 210 and 195 °C. The obtained films were cut in 10 cm^2^ sheets and accurately weighed. The thickness of the films were measured by using a manual digital micrometer (Mitutoyo, Japan) and measurements were performed in different parts of the film. The film thickness ranged between 50 and 65 µm and the photoinitiators concentration measured in the LDPE films were 9.5 × 10^2^ ± 1.3 × 10^2^ mg∙kg^−1^ and 6.7 × 10^2^ 1.9 × 10^2^ mg∙kg^−1^, for 4-MBP and EDB, respectively. 

Migration tests were as follows: the additivated LDPE films were introduced in a light protected tube, containing 20 mL of a food simulant at the selected temperature. At different previously pre-set times, 0.5 mL of the simulant was removed from the tube, filtered and injected into the HPLC-DAD system (Hewlett-Packard, Waldbronn, Germany) to determine the exact concentration of migrant at each time interval. The total time of migration assays were the following, 8 h, 2, days 7 days and 210 days at 40, 20, 4 and −18 °C, respectively for 4-MBP. In the case of EDB, the total time of migration tests were the same except at 20 °C which was for one day. At the end of each experiment, the LDPE film was removed from the tube, extracted with 20 mL of acetonitrile for 24 h at 70 °C, and analysed by HPLC-DAD to determine the photoinitiator remnant in the polymer. All assays were done in duplicate.

The HPLC-DAD method was based on the method developed by Lago et al. (2014) with a minor modification in the mobile phase gradient: after the photoinitiator was eluted, the percentage of acetonitrile was raised to 100% for 1 min and then held for 2 min [28].

## 4. Conclusions

The migration kinetics of two photoinitiators specifically EDB and 4-MBP into food simulants at different temperatures, (−18, 4, 20 and 40 °C) were studied. To the best of our knowledge very scarce information on migration kinetics at freezing temperatures has been reported in the literature.

A HPLC-DAD method was optimized to determine the photoinitiators in the food simulants. A mathematical model based on Fick’s second law was applied to calculate the diffusion coefficients and the values obtained varied between 5.5 × 10^−13^ and 3.1 × 10^−8^ cm^2^s^−1^. The diffusion process was significantly affected by the temperature, the percentage of ethanol and pH of the food simulants. RMSE values were in all cases ≤ 9%, showing good fitting between the estimated and experimental data, thus this model can be used to predict the migration of these photoinitiatros into the food simulants.

## Figures and Tables

**Table 1:** Coefficients of diffusion (*D_P_*), partition (*K_P/F_*) and Root-Mean-Square Error (RMSE) of 4-methyl benzophenone (4-MBP) and ethyl-4-(dimethylamino) benzoate (EDB).

Food/Food Simulant	Temperature (°C)	4-MBP			EDB		
		*D_P_* (cm^2^∙s^−1^)	*K_P/F_* (*w/v*)	RMSE (%)	*D_P_* (cm^2^∙s^−1^)	*K_P/F_* (*w/v*)	RMSE (%)
**Water**	4	1.0 × 10^−10^	309.0	3.4	2.6 × 10^−10^	60.3	3.9
	20	1.5 × 10^−10^	365.5	3.0	8.9 × 10^−10^	47.1	2.2
	40	3.2 × 10^−10^	302.5	2.5	3.0 × 10^−9^	119.4	3.5
**3% Acetic acid (*w/v*)**	4	1.9 × 10^−10^	279.7	3.5	4.6 × 10^−10^	22.6	2.2
	20	2.4 × 10^−10^	407.0	3.1	2.1 × 10^−9^	22.2	4.3
	40	4.1 × 10^−10^	353.3	3.9	6.8 × 10^−9^	20.0	3.4
**10% Ethanol (*v/v*)**	4	1.3 × 10^−10^	257.7	6.9	3.2 × 10^−10^	42.7	3.7
	20	4.9 × 10^−10^	239.8	3.8	1.3 × 10^−9^	38.5	5.1
	40	1.7 × 10^−9^	148.6	3.9	5.1 × 10^−9^	30.2	4.7
**20% Ethanol (*v/v*)**	4	1.6 × 10^−10^	148.2	5.5	3.9 × 10^−10^	18.2	4.4
	20	7.8 × 10^−10^	82.7	3.7	1.5 × 10^−9^	23.5	4.5
	40	1.9 × 10^−9^	78.9	6.3	1.2 × 10^−8^	13.0	2.5
**50% Ethanol (*v/v*)**	−18	1.5 × 10^−12^	13.9	4.7	5.5 × 10^−13^	25.4	2.1
	4	1.3 × 10^−9^	3.3	4.3	4.7 × 10^−10^	5.1	5.7
	20	5.5 × 10^−9^	3.5	4.8	2.1 × 10^−9^	3.9	6.0
	40	1.1 × 10^−8^	4.9	8.9	8.5 × 10^−9^	<2.3 *	5.4
**95% Ethanol (*v/v*)**	−18	2.8 × 10^−12^	20.6	3.6	7.8 × 10^−13^	37.1	3.4
	4	1.3 × 10^−9^	<1.5 *	3.6	4.0 × 10^−10^	4.9	4.1
	20	4.5 × 10^−9^	1.9	3.3	2.8 × 10^−9^	<1.5 *	4.0
	40	3.1 × 10^−8^	2.6	3.8	6.4 × 10^−9^	<1.4 *	6.3

* The method quantification limit (LOQ = 0.025 mg·L^−1^) does not allow the estimation of lower values of *K_P/F_*.

**Table 2:** Experimental *D*_0_, *E_A_* and R^2^ values calculated with equation 3 for 4-methyl benzophenone (4-MBP) and ethyl-4-(dimethylamino) benzoate (EDB).

Food/Food Simulant	Migrant	*E_A_* (kJ∙mol^−1^)	*D*_0_ (cm^2^∙s^−1^)	R^2^
**H_2_O**	4-MBP	22.35	1.63 × 10^−6^	0.98009
	EDB	48.62	3.89 × 10^−1^	0.99877
**3% Acetic Acid**	4-MBP	15.75	1.67 × 10^−7^	0.95748
	EDB	53.81	6.94	0.98835
**10% Ethanol**	4-MBP	52.11	8.76 × 10^−1^	0.99722
	EDB	55.05	8.02	0.99752
**20% Ethanol**	4-MBP	48.70	2.84 × 10^−1^	0.96626
	EDB	69.20	3.91 × 10^3^	0.99036
**50% Ethanol**	4-MBP	42.57	1.59 × 10^−1^	0.95148
	EDB	58.09	4.32 × 10^1^	0.99733
**95% Ethanol**	4-MBP	63.32	1.03 × 10^3^	0.99003
	EDB	60.73	1.11 × 10^2^	0.91564
**Theoretical Prediction** **(Piringer)**	4-MBP	86.9	1.86 × 10^7^	-
	EDB		1.93 × 10^7^	

**Table 3:** Summary of the main properties of 4-methyl benzophenone (4-MBP) and ethyl-4-(dimethylamino) benzoate (EDB). Mw: molecular weight; Mp: melting point; Bp: boiling point; PI: photoinitiator; a: experimental; b: estimated. Data was extracted from SciFinder database in 2015.

Structure	CAS nr.	Common Name	Mw	Log K_o/w_	Mp (°C)	Bp (°C)	PI Type
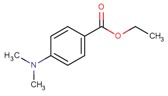	10287-53-3	EDB	193.24	2.51 ^b^	63.50 ^a^	296.50 ^b^	Amine synergist
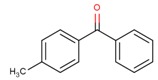	134-84-9	4-MBP	196.24	3.69 ^b^	59.50 ^a^	328.10 ^b^	II

## References

[1] Castle L., Bradley E.L., Nollet L.N.L., Toldrá F. (2010). Residues of food contact materials. Handbook of Dairy Foods Analysis.

[2] Simoneau C., Picó Y. (2008). Food contact materials. Comprehensive Analytical Chemistry. Food Contaminants and Residue Analysis.

[3] Lau O.W., Wong S.K. (2000). Contamination in food from packaging material. J. Chromatogr. A.

[4] Brandsch J., Mercea P., Rüter M., Tosa V., Piringer O. (2002). Migration modelling as a tool for quality assurance of food packaging. Food. Addit. Contam..

[5] Helmroth I.E., Rijk R., Dekker M., Jongen W. (2002). Predictive modelling of migration from packaging materials into food products for regulatory purposes. Trends Food Sci. Technol..

[6] Helmroth I.E., Dekker M., Hankemeier T. (2002). Influence of solvent absorption on the migration of Irganox 1076 from LDPE. Food Addit. Contam..

[7] Petersen J.H., Trier X.T., Fabech B. (2005). Mathematical modelling of migration: A suitable tool for the enforcement authorities?. Food Addit. Contam..

[8] Sanches Silva A.T., Sendón García R., Cooper I., Franz R., Paseiro P. (2006). Compilation of analytical methods and guidelines for the determination of selected model migrants from plastic packaging. Trends Food. Sci. Technol..

[9] Sanches Silva A.T., Cruz J.M., Sendón García R., Franz R., Paseiro P. (2007). Kinetic migration studies from packaging films into meat products. Meat Sci..

[10] Sanches Silva A.T., Cruz Freire J.M., Sendón R., Franz R., Paseiro Losada P. (2009). Migration and diffusion of diphenylbutadiene from packages into foods. J. Agric. Food Chem..

[11] Sanches Silva A.T., Cruz Freire J.M., Paseiro Losada P. (2010). Study of the diffusion coefficients of diphenylbutadiene and triclosan into and within meat. Eur. Food Res. Technol..

[12] (2011). Commission Regulation (EU) No 10/2011 of 14 January 2011 on plastic materials and articles intended to come into contact with food. Off. J. Eur. Union.

[13] (2004). Commission Regulation (EU) No 1935/2004 of 27 October 2004 on materials and articles intended to come into contact with food. Off. J. Eur. Union.

[14] Bustos J., Martin P., Lago M.A., Sendón R., Rodríguez Bernaldo de Quirós A., Puga M.A., Basadre Pampín M.I., Sánchez J.J. GC/MS screening of ink compounds (photoinitiators) in food packaging. Proceedings of the 5th International Symposium on Food Packaging: Scientific Developments Supporting Safety and Innovation.

[15] RASFF Portal. https://webgate.ec.europa.eu/rasff-window/portal/.

[16] Lago M.A., Rodríguez-Bernaldo de Quirós A., Sendón R., Bustos J., Nieto M.T., Paseiro P. (2015). Photoinitiators: A food safety review. Food Addit. Contam. A.

[17] Rodríguez-Bernaldo de Quirós A., Paseiro-Cerrato R., Pastorelli S., Koivikko R., Simoneau C., Paseiro-Losada P. (2009). Migration of photoinitiators by gas phase into dry foods. J. Agric. Food Chem..

[18] Van Den Houwe K., Van Heyst A., Evrard C., Van Loco J., Bolle F., Lynen F., Van Hoeck E. (2016). Migration of 17 photoinitiators from printing inks and cardboard into packaged food—Results of a Belgian market survey. Packag. Technol. Sci..

[19] Liu P., Zhao C., Zhang Y., Chen Y. (2016). Simultaneous determination of 10 photoinitiators in milk by solid-phase microextraction coupled with gas chromatography/mass spectrometry. J. Food Sci..

[20] Crank J. (1975). The Mathematics of Diffusion.

[21] Piringer O.G. (1994). Evaluation of plastics for food packaging. Food Addit. Contam..

[22] Sanches-Silva A., Pastorelli S., Cruz J.M., Simoneau C., Castanheira I., Paseiro-Losada P. (2008). Development of a method to study the migration of six photoinitiators into powdered milk. J. Agric. Food Chem..

[23] Simoneau C. Applicability of Generally Recognised Diffusion Models for the Estimation of Specific Migration in Support of EU Directive 2002/72/EC. http://publications.jrc.ec.europa.eu/repository/bitstream/111111111/14935/1/reqno_jrc59476_mathmod_v10_cs_2010_09_24_final.pdf%5B1%5D.pdf.

[24] Lago Crespo M.A. (2016). Printing Inks for Food Packaging. Study of the Key Parameters in the Migration of Phthoinitiators. Ph.D. Thesis.

[25] Brandsch J., Mercea P., Piringer O., Piringer O.G., Baner A.L. (2000). Possibilities and limitations of migration modelling. Packaging Materials for Food. Plastic Barrier Function, Mass Transport, Quality Assurance and Legislation.

[26] Maia J., Rodríguez-Bernaldo de Quirós A., Sendón R., Cruz J.M., Seiler A., Franz R., Simoneau C., Castle C., Driffield M., Mercea P. (2016). The Determination of key diffusion and partition parameters and their use in migration modelling of benzophenone from low density polyethylene (LDPE) into different foodstuffs. Food Addit. Contam. A.

[27] (1985). Council Directive (EU) No 85/572/ECC of 19 December 1985 laying down the list of simulants to be used for testing migration of constituents of plastic materials and articles intended to come into contact with foodstuffs. Off. J. Eur. Comm..

[28] Lago M.A., Rodríguez-Bernaldo de Quirós A., Sendón R., Bustos J., Santillana M.I., Paseiro P. (2014). Simultaneous chromatographic analysis of photoinitiators and amine synergists in food contact materials. Anal. Bioanal. Chem..

